# Ultrasonographic measurement of glottal area: A potential biomarker study in young Normophonic adults

**DOI:** 10.1007/s00405-025-09739-5

**Published:** 2025-10-18

**Authors:** Santosh Rai, Anika Tiku, Anjali Menon, Divya Seth, Gagan Bajaj

**Affiliations:** 1 Department of Radiodiagnosis & Imaging, Kasturba Medical College Mangalore, Manipal Academy of Higher Education, Manipal, India; 2 Department of Audiology and Speech Language Pathology, Kasturba Medical College Mangalore, Manipal Academy of Higher Education, Manipal, India; 3 Department of Speech Language Pathology, All India Institute of Speech and Hearing, Mysore, India

**Keywords:** Glottal area, Vocal folds, Ultrasound, Acoustic analysis, Voice

## Abstract

**Purpose:**

Advancing from previous work on ultrasonography (USG)-aided measures including vocal fold morphology and symmetry, vocal fold length (VFL), vocal fold displacement velocity (VFDV) and its acoustic correlates [1], the present study broadened the investigation by exploring additional parameters such as glottal area during the inhalation phase of quiet breathing (QB), its relationship with VFL, VFDV and acoustic measures such as fundamental frequency (*f*_*0*_), jitter, cepstral peak prominence (CPP) in young normophonic adults (25–30 years).

**Method:**

A total of 117 participants were recruited from our hospital, and USG was performed on them across tasks like QB and vowel phonation (/a/ and /i/). A detailed voice evaluation including perceptual analysis of voice using GRBAS (Grade Roughness Breathiness Asthenia Scale) to grade the voice quality and acoustic analysis using Praat software to measure voice parameters was carried out.

**Results:**

The mean glottal area during the inhalation phase of quiet breathing (QB) was found to be 1.407 cm² (SD = 0.086 cm²) in males and 1.106 cm² (SD = 0.080 cm²) in females. Significant correlation was found between glottal area and VFL, VFDV and CPP values during /i/ phonation.

**Conclusion:**

Based on these results, we recommend that USG-aided estimation of glottal area can serve as a potential vocal fold biomarker for clinicians and researchers in laryngology.

## Introduction

Ultrasonography (USG) is gaining popularity as a promising non-invasive diagnostic tool [2] utilized by health care professionals like laryngologists, radiologists and speech-language pathologists (SLPs) to assess functioning of the laryngeal system [1, 3]. Recent studies emphasize USG’s reliability in assessing vocal folds and its ability to provide more consistent methods for evaluating laryngeal functions [2, 4]. USG offers several diagnostic and functional advantages, including improved sensitivity and greater accessibility compared to other well-established techniques [5]. It serves as a convenient and accessible alternative, with its non-invasive nature promoting safer use and improved patient tolerance [1]. USG allows patients to remain in a relaxed position during examinations, avoiding the discomfort often associated with other techniques such as flexible laryngoscopy, MRI, or CT, which may require invasive procedures, restricted positioning, or longer scan times. While imaging methods like stroboscopy, video kymography and high-speed digital imaging offer direct visualization of the vocal folds, USG could be particularly advantageous in settings where patient comfort is essential [6], repeated tests are warranted [7] with minimal risk of infection, and cost-effectiveness [1, 8].

USG can be used to measure both anatomical and physiological aspects of the laryngeal system such as mucosal waves, muscle length, and speech valve adaptation [9]. It is an effective supplementary or alternative method to assess larynx as it provides high visualization rates of thyroid and cricoid structures, as supported by research [1]. Moreover, USG is a reliable tool with excellent sensitivity and specificity for diagnosing vocal cord palsy [10]. Studies show that USG is more effective than MRI and CT in evaluating vocal fold motility [11]. It can be used for preoperative assessment of vocal fold mobility by assessing the inter-arytenoid distance or examining recorded video observations [12]. Additionally, it shows high sensitivity in detecting recurrent laryngeal nerve palsy in patients after thyroidectomy and has been utilized as a reference for evaluating vocal fold areas and angles in healthy individuals [13, 14].

While the safety, non-invasiveness, and non-radioactive nature of USG offer significant clinical advantages, further research is required to establish standardized protocols and enhance its clinical utility for evaluation of vocal cords. The current lack of vocal fold related normative data, on USG, presents a challenge to its widespread clinical application in patient evaluation. Recently, a landmark study was conducted to facilitate the seamless translation of USG into clinical practice for the comprehensive evaluation of laryngeal functions [2]. To identify the high priority areas, two formal meetings were held among an international USG working group [2]. A framework on stakeholders, protocols and training, and evidence-base and metrics was provided. Besides validity and reliability, a key priority area that was identified included establishing normative data for vocal fold biomarkers using USG across individuals belonging to different age groups as well different conditions. The study also emphasized that, while some preliminary data exist on the echo intensity and thickness of the tongue and submental muscles, as well as hyoid bone movement in both aging and healthy young adults, there remains a lack of established standards for evaluating vocal fold biomarkers [2].

Given the advantages of USG, its potential for assessing vocal fold biomarkers, and the recommendations from the international USG working group to establish age and condition specific normative data, studies have sought to investigate various vocal fold parameters like vocal fold displacement velocity (VFDV) and vocal fold length (VFL) using USG among young normophonic adults [1]. VFDV reflects a component of mucosal wave velocity and has been suggested as an alternative method for assessing vocal fold stiffness, which plays a crucial role in the generation of mucosal waves [15–17]. VFL on the other hand, helps in the analysis of vocal fold functioning and is determined by measuring the length of the vocal fold from the anterior commissure of the arytenoid cartilage to the edge of the vocal process [18]. Rai & colleagues [1] characterized vocal fold morphology, symmetry, and gender-specific differences, as well as task-specific USG -aided data on VFL and VFDV, in young normophonic adults aged 18–30 years. Participants in the study underwent USG across three tasks including quiet breathing (QB),/*a*/and/*i*/phonation. Additionally, acoustic analysis was conducted to explore the relationship between USG and acoustic measures. The findings of the study revealed that males had longer vocal folds than females. Overall greater vocal fold velocities were observed in/*a*/phonation, followed by/*i*/phonation, with the lowest velocity observed in the QB task. The obtained norms were considered a strong preliminary step toward the quantitative benchmarking of vocal fold behaviour in young adults. While Rai & colleagues [1] demonstrated the initial utility of USG in assessing vocal folds by identifying at least two potential biomarkers - VFL and VFDV, the study further emphasized its potential for expanding the evaluation to include additional biomarkers and widening the efforts for other population demographics.

Building on this foundation, the present study examines glottal area as another USG-aided critical vocal fold biomarker. The glottal area is essential for voice production, serving as the main source of voiced sound [19]. Glottal area refers to the space between the vocal folds, where airflow from the lungs is controlled to produce fundamental frequency (*f*_0_) and harmonics that shape speech [19]. Glottal area waveform analysis, which represents the variation in relative glottal area over time within a typical glottal cycle, serves as a critical tool for evaluating vocal fold vibration and function [20]. This analysis is crucial in clinical settings for patients with persistent hoarseness following voice therapy, as it helps assess therapy effectiveness and guides clinicians in optimizing treatment or considering surgical intervention [21]. Studies have shown that glottal area waveform analysis before and after phonomicrolaryngeal surgery in patients with vocal pathologies such as polypoid degeneration, vocal fold polyps, sulcus vocalis, Reinke’s edema, and cysts reveals significant post-surgical changes, including increased maximum glottal area and enhanced closing and opening rates [22]. These findings highlight its value in quantifying vocal fold behaviour and assessing treatment outcomes [22]. Additionally, glottal area evaluation is instrumental in assessing the severity of glottic incompetence in patients with unilateral or bilateral vocal cord paralysis [23]. Recent advancements have demonstrated that USG can provide a non-invasive method to assess glottal area, vocal fold movement, and airway patency in laryngeal pathologies, further expanding USG’s clinical utility [5]. Furthermore, the association between glottal area and acoustic measures has gained significant attention from researchers [24–27].

Acoustic measures, such as perturbation indices (e.g., jitter and shimmer), are valuable in detecting voice pathologies and are often associated with incomplete glottal closure. This, in turn, is linked to variations in glottal area and irregular vocal fold movement [25]. Changes in the open quotient (OQ) and speed quotient (SQ), which reflect the timing of vocal fold opening and closure, have a direct impact on the glottal area waveform, in turn affecting the acoustic properties of the voice, such as pitch and intensity [24, 26]. Research on glottal area changes and their impact on (*f*_0_) has been crucial in understanding voice quality. Studies using open quotient as an indirect measure of glottal area have shown no correlation between open quotient and *f*_0_ in male speakers, while female speakers exhibit an increase in open quotient with rising *f*_0_ [24]. Such gender-based differences may stem from variations in laryngeal mechanisms, including vocal fold length, thickness, and muscular tension [24]. Woo (1996) observed significant gender and age differences in glottal area waveform, with young women often displaying a triangular or vase-shaped glottis, while young men, elderly men, and elderly women exhibited a spindle-shaped configuration. Additionally [18], reported age and sex-related differences in glottal area changes concerning frequency. Therefore, estimating the glottal area is crucial for establishing a baseline in clinical assessments, identifying pathological voice functions, and guiding treatment planning and monitoring [8, 20].

Recognizing that the glottal area could serve as a potential biomarker, and as part of an ongoing effort to identify vocal fold biomarkers using USG, the present study aimed to profile the USG-assisted glottal area among young normophonic individuals. The specific objectives included measuring the glottal area using USG and evaluating its association with acoustic parameters such as (f₀), jitter, and cepstral peak prominence (CPP). Additionally, the study explored the correlation of the glottal area with USG-derived measures of VFL and VFDV, as well as its relationship with age and gender.

## Materials and methods

The present study was conducted between August 2022 and April 2024. The study received approval from the Institutional Ethical Committee (IEC XXX XXX 07/2022/286) in compliance with the ethical guidelines for human research set forth in the Second Declaration of Helsinki. A written informed consent was obtained from all the participants prior to the enrolment in this study.

### Participants

Individuals with normal phonatory functions on GRBAS (Grade Roughness Breathiness Asthenia Scale) [28], within the age range of 20–35 years were recruited for the study from the hospital including individuals with various medical conditions that are unrelated to voice or unlikely to have any influence on voice or laryngeal function, students, staff and patient’s caregivers. The sample size of 117 participants was calculated using a standard deviation of 4, at 1% alpha error and at 95% confidence level, based on the reference article [29]. The sample size was calculated using the given formula:

$$\left[n=Z_{1-n/2^{2\;}}SD\right]$$

Individuals with a history of smoking, chronic voice abuse or misuse, vocal fold irregularities or conditions such as nodules, polyps, or paralysis, as well as those with diagnosed dysphonia, upper respiratory disorders, thyroid-related pathologies, or reflux conditions were excluded from the study.

### Procedure

**Fig. 1:**
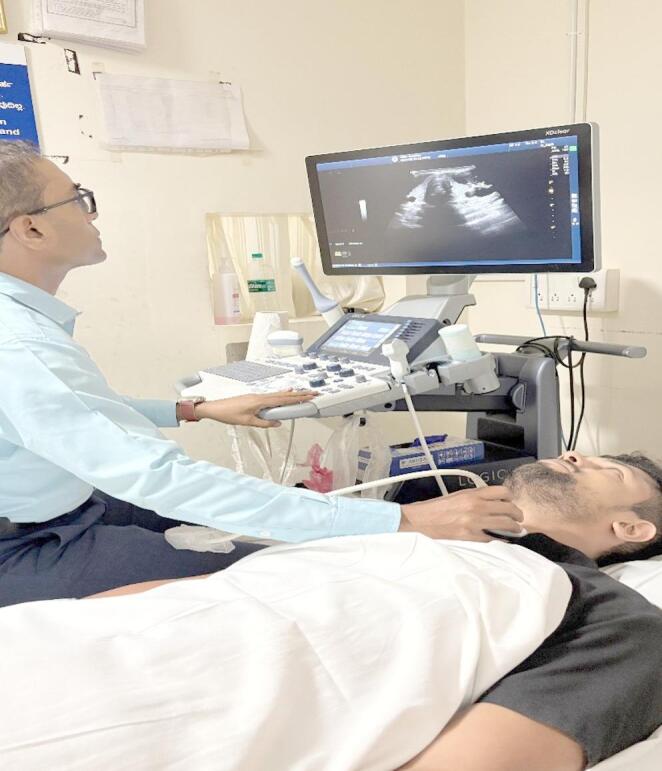
USG Recording

The current study adhered to a procedure similar to that employed by Rai & colleagues [1]. A detailed case history was taken from the participants keeping in mind the inclusion and exclusion criteria and screening was done using GRBAS scale [28]. The USG was done using a transcutaneous B mode ultrasound, performed by a qualified radiologist with more than 15 years of experience and a junior resident with at least 2 years of training in ultrasonography (Fig. 1). Participants were positioned in the supine posture with a pillow placed under the neck to facilitate neck extension, thereby enhancing the visualization of the vocal folds and surrounding laryngeal structures. Imaging was performed using a linear array transducer (7–12 MHz) placed horizontally on the lateral aspect of thyroid lamina, following the protocol described by Rai & colleagues [1]. The focus, scale and gain settings were adjusted for optimal visualization of the vocal folds. To ensure consistency in probe placement and imaging plane, anatomical landmarks such as the thyroid cartilage and arytenoids were identified, with the mid-glottic level used as a reference point for glottal area measurements [29]. Regarding frame selection, still images were captured at the point of maximum vocal fold abduction during inhalation, based on visual inspection of dynamic ultrasound recordings. This approach was used consistently across participants to ensure standardization of frame selection.

**Fig. 2:**
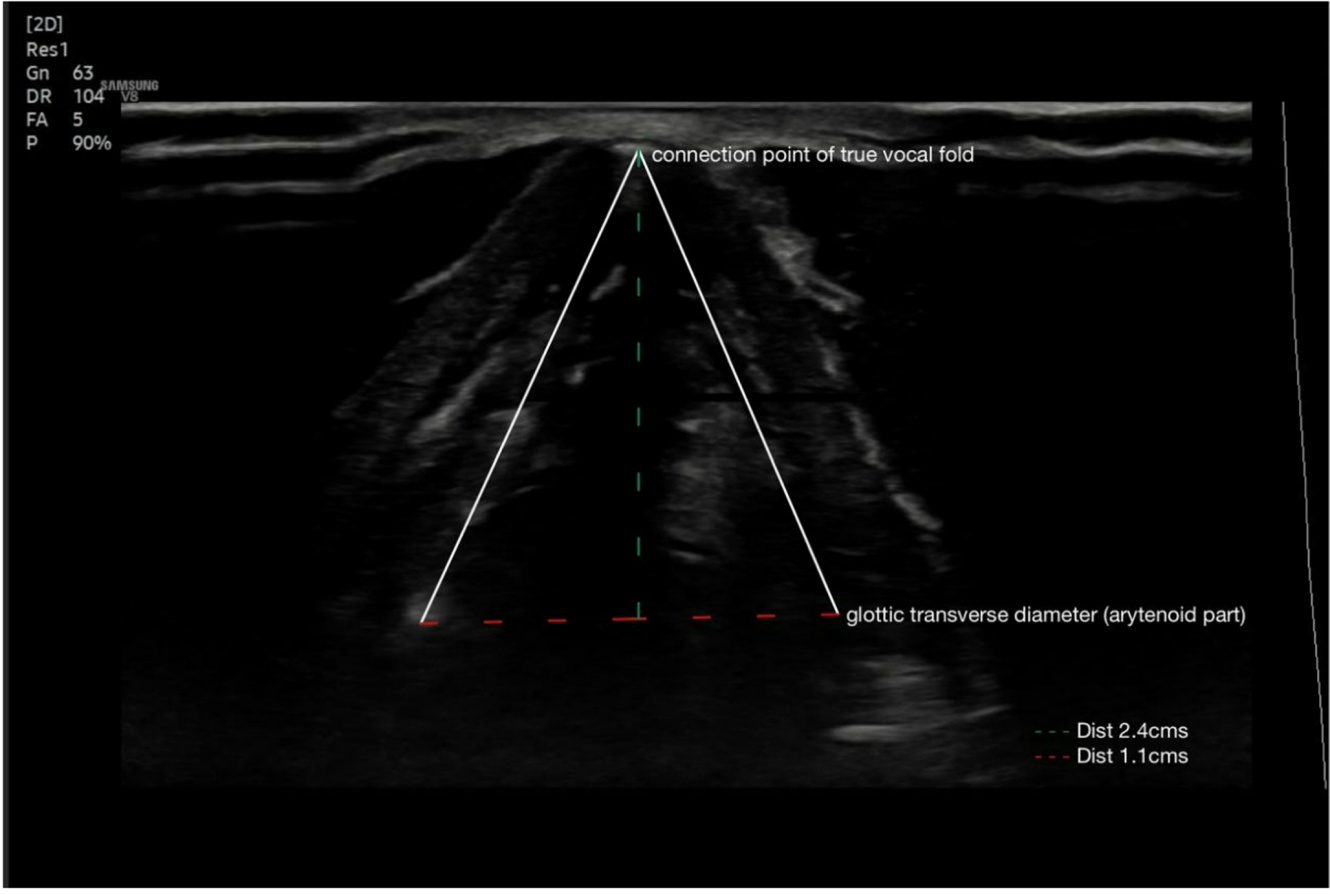
Glottal Area Measurement

Vocal fold morphology and symmetry were assessed based on Rai et al.’s criteria [1]. To measure the glottal area, participants were instructed to perform quiet breathing, and a probe was placed at the mid glottic level which was identified using thyroid cartilage and arytenoids as landmarks [29]. The image was deemed adequate when the vocal folds were visible bilaterally, anterior commissure seen anteriorly and arytenoids seen on both sides posteriorly. Drawing inspiration from the glottal area estimation from Bright & colleagues [29],during inhalation, when the true vocal folds were seen as abducted, three lines were drawn, two extending from the anterior commissure to either sides of the arytenoids (sides of the triangle) and another line made between the two arytenoids (base). Then a hypothetical perpendicular line was drawn extending from the anterior commissure to the basal line below (height) [29]. The region of interest was delineated by tracing along the clearly visualized margins of the vocal folds, identified by the distinct air, soft tissue interface. This high-contrast boundary enabled accurate delineation of the glottal contour, allowing for reliable area estimation. Hence, a triangle was formed with base and height as mentioned earlier. The distance between them was then measured in centimetres. Consistent imaging settings and anatomical landmarks were maintained to ensure reproducibility across measurements. A sample of USG aided estimation glottal area has been depicted in the Fig. 2. Formula for area under triangle was used, i.e. ½ × base × height to measure the glottal area. During the procedure, other USG-aided measures such as VFL and VFDV, were also recorded, and the data were entered on an observation sheet. Both these measurements were done with a frame rate of 7 Hz pulse and a repetition rate of 10,000 Hz using the colour mode. The vocal fold displacement velocity (VFDV) was calculated using the following formula: [VFDV = Displacement (d)/Time (t)] [1]. It is important to note that the measured length of structures on ultrasound may differ slightly from their true anatomical length due to factors like beam geometry, variations in propagation speed, and the angle of insonation. To minimize these differences, appropriate focus placement was ensured during scanning. Additionally, the ultrasound system was calibrated, and image acquisition settings were standardized throughout the study to enhance measurement consistency and reproducibility. All the USG-derived measurements were obtained during the inhalation phase of quiet breathing (QB).

Acoustic analysis was carried out using Praat software (Version 6.2.14) on a (HP 19 ka) monitor, while the audio samples were captured using a (Shure C606) microphone. The recordings were done in an acoustically treated recording room, wherein participants were made to sit upright, and a microphone was placed at 5 cm from their mouth at an angle of 45°. Participants were asked to phonate vowels (/a/,/i/) and maintain the phonation for 3–5 s, with three trials recorded for each vowel. The best sample [30] was chosen for the analysis of acoustic measures, including *f*_0_, jitter, and CPP.

### Data analysis

Vocal fold morphology and symmetry were classified with a rubric that categorized morphology into adequately visualized, poorly visualized, or not visualized, and symmetry into Grades I to III movement based on mobility and position [1]. “Grade I” indicates that both vocal folds move symmetrically and fully during abduction and adduction, with no delay, restriction, or asymmetry observed. “Grade II” represents mild asymmetry in the movement or position of one or both vocal folds, with some preserved movement, which may include delayed onset, reduced range of motion, or a phase difference. “Grade III” indicates complete absence of movement in one or both vocal folds, with marked asymmetry, suggestive of vocal fold paralysis, fixation, or severe neuromuscular impairment [1].

Statistical analysis of the quantitative data was conducted using SPSS version 25. The mean and standard deviation of the USG measurements, VFL, VFDV, and glottal area was subjected to descriptive statistics. An Independent samples t-test was used to determine the gender and age-specific differences of USG-aided glottal area measures. Further, Spearman’s correlation was used to study the relationship between glottal area and other parameters such as VFL, VFDV and acoustic measures such as *f*_0_, jitter and CPP. A *p*-value of less than 0.05 was considered statistically significant.

## Results

The present study aimed to profile USG aided glottal area along with vocal fold morphology and symmetry, VFL, and VFDV among young normophonic adults. The study also examined the association of glottal area with VFL, VFDV, and acoustic parameters such as *f*_0_, jitter, and CPP. Additionally, the study explored gender and age-related variations in glottal area to determine the presence of any demographic differences.

### Vocal fold morphology and symmetry

Based on the visibility of laryngeal structures such as the cricoid cartilage, thyroid cartilage, false vocal folds, true vocal folds, anterior commissure, and posterior commissure of the vibrating vocal folds, the anatomical visualization of vocal structures was rated as ‘adequately visualized’ for all participants (100%). Regarding vocal fold symmetry, based on the observed movement patterns, the physiological condition of the vocal folds in all participants (100%) was classified as ‘Grade I (Normal mobility)’.

### Glottal area in males and females during quiet breathing (QB)

The mean glottal area, as shown in Table 1, during the inhalation phase of QB was found to be significantly higher in males (M = 1.407 cm², SD = 0.086 cm²) compared to females (M = 1.106 cm², SD = 0.080 cm²) [*t*(115) = 17.112, *p* < 0.001].

**Table 1:** Descriptive statistics of glottal area in males and females

Gender	*N*	Mean (cm^2^)	SD (cm^2^)	t-stat	*p*-value
Male	85	1.407	0.086	17.112	< 0.001**
Female	32	1.106	0.080		

### Gender-Specific correlation of glottal area with age

Spearman’s correlation analysis revealed a non-significant relationship between USG-aided glottal area measures and age for both male [(*ρ*(83) = − 0.10, *p* = 0.34)] and female participants [(*ρ* [30] = − 0.21, *p* = 0.25)], suggesting that the young normophonic group (aged 20–35 years) was relatively homogeneous with respect to age and glottal area.

### Vocal fold length (VFL)

**Fig. 3:**
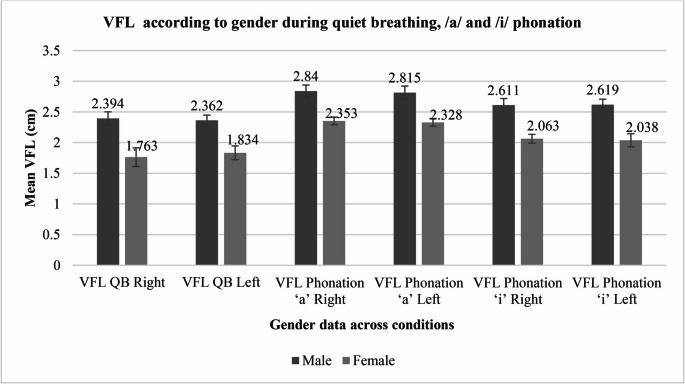
Gender Differences in VFL Across Left and Right sides during QB and Phonation of/a/and/i/

The mean VFL during QB was 2.200 cm (SD = 0.308 cm) on the right side and 2.218 cm (SD = 0.254 cm) on the left side. During phonation of the vowel/a/, the mean VFL was 2.707 cm (SD = 0.236 cm) on the right and 2.682 cm (SD = 0.239 cm) on the left. For phonation of the vowel/i/, the mean VFL measured 2.461 cm (SD = 0.265 cm) on the right and 2.460 cm (SD = 0.277 cm) on the left. These findings are illustrated in Fig. 3. The data also revealed significant differences in VFL between males and females across all conditions (QB,/a/and/i/phonation) and sides (left & right) (*p* < 0.001), as presented in Table 2, with males consistently having higher VFL values than females.

**Table 2:** Gender differences in VFL across left and right sides during QB and phonation of/a/and/i/

Variable	Sex	*N*	Mean (cm)	SD (cm)	t-stat	*p*-value
VFL QB Right	Male	85	2.394	0.108	25.033	< 0.001**
	Female	32	1.763	0.152		
VFL QB Left	Male	85	2.362	0.086	27.141	< 0.001**
	Female	32	1.834	0.112		
VFL Phonation/a/Right	Male	85	2.840	0.098	26.194	< 0.001**
	Female	32	2.353	0.062		
VFL Phonation/a/Left	Male	85	2.815	0.107	24.074	< 0.001**
	Female	32	2.328	0.063		
VFL Phonation/i/Right	Male	85	2.611	0.108	26.595	< 0.001**
	Female	32	2.063	0.071		
VFL Phonation/i/Left	Male	85	2.619	0.089	29.691	< 0.001**
	Female	32	2.038	0.107		

Note. ^*1*^*VFL: Vocal Fold Length;* ^*2*^*QB: Quiet Breathing*

### Vocal fold displacement velocity (VFDV)

**Fig. 4:**
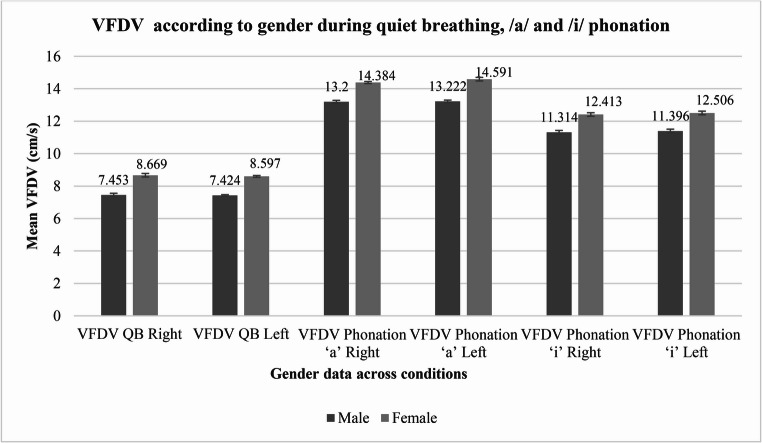
Gender Differences in VFDV Across Left and Right Sides During QB and Phonation of/a/and/i/

The mean VFDV (cm/s) during QB was 7.785 (SD = 0.554) on the right side and 7.744 (SD = 0.527) on the left side. During phonation of the vowel/a/, the mean VFDV was 13.524 (SD = 0.536) on the right and 13.597 (SD = 0.619) on the left. For phonation of the vowel/i/, the mean VFDV measured 11.615 (SD = 0.505) on the right and 11.7 (SD = 0.509) on the left. These findings are depicted in Fig. 4. Additionally, the data presented in Table 3 shows significant differences in VFDV between males and females across all conditions and sides (*p* < *0.001*), with females consistently having higher VFDV values than males.

**Table 3:** Gender differences in VFDV across left and right sides during QB and phonation of/a/and/i/

Variable	Sex	*N*	Mean (cm/s)	SD (cm/s)	t-stat	*p*-value
VFDV QB Right	Male	85	7.453	0.102	−56.442	< 0.001*
	Female	32	8.669	0.109		
VFDV QB Left	Male	85	7.424	0.045	−118.385	< 0.001*
	Female	32	8.597	0.054		
VFDV Phonation/a/Right	Male	85	13.20	0.085	−72.062	< 0.001*
	Female	32	14.384	0.063		
VFDV Phonation/a/Left	Male	85	13.222	0.076	−76.525	< 0.001*
	Female	32	14.591	0.109		
VFDV Phonation/i/Right	Male	85	11.314	0.116	−46.401	< 0.001*
	Female	32	12.413	0.110		
VFDV Phonation/i/Left	Male	85	11.396	0.111	−48.024	< 0.001*
	Female	32	12.506	0.113		

Note: ^*3*^*VFDV: Vocal Fold Displacement Velocity*

### Correlation between USG-aided glottal area measurement and VFL, VFDV

Spearman’s correlation analysis was conducted to examine the relationship between USG-aided glottal area measurements and VFL, VFDV. The results, as shown in Table 4, revealed a significant moderate positive correlation between glottal area and VFL on the right side [*ρ*(115) = 0.55, *p* < 0.001], and a significant moderate-to-strong positive correlation on the left side [*ρ*(115) = 0.59, *p* < 0.001] under QB conditions. Additionally, a significant strong negative correlation was observed between glottal area and VFDV on both the right [*ρ*(115) = −0.65, *p* < 0.001] and left sides [*ρ*(115) = −0.63, *p* < 0.001].

**Table 4:** Correlation between USG-aided glottal area measurement and VFL and VFDV

	Glottal area (cm^2^)	
VFL & VFDV	*R* (ρ) values	*p* values
VFL Right QB	0.555	< 0.001
VFL Left QB	0.586	< 0.001
VFDV Right QB	−0.659	< 0.001
VFDV Left QB	−0.639	< 0.001

### Correlation between USG-aided glottal area measurement and acoustic measures

Due to logistic constraints, only 50 out of 117 recordings (42.7%) were obtained for acoustic analysis. Spearman’s correlation analysis was conducted to examine the relationship between USG-aided glottal area measurements and acoustic measures, including *f*_0_, jitter, CPP full, and CPP with voice detection during the phonation of/a/and/i/. A significant correlation was obtained between CPP with voice detection and glottal area during/i/phonation as reported in Table 5. All other correlations, including those between glottal area and *f*_0_, jitter, CPP full for/a/and/i/phonation, as well as CPP with voice detection for/a/phonation were statistically non-significant.

**Table 5:** Correlation between glottal Area(cm^2^) and acoustic measures

	Glottal area	
Acoustic Parameters	*R* (ρ) values	*p* values
*f*_0_ (/a/)	−0.132	0.362
Jitter (/a/)	0.237	0.097
CPP full (/a/)	0.116	0.424
CPP with Voice detection (/a/)	0.232	0.105
*f*_0_ (/i/)	−0.175	0.223
Jitter (/i/)	0.12	0.407
CPP full (/i/)	0.208	0.146
CPP with Voice detection/i/)	0.316	0.025

Note: ^*4*^*f*_*0*_: *Fundamental Frequency;* ^*5*^*CPP: Cepstral Peak Prominence*

## Discussion

USG has recently gained popularity due to its ability to detect both typical and atypical laryngeal functions, offering several advantages over traditional methods, including non-invasive nature [2], good patient tolerance, low cost, safety, speed, accessibility, painlessness, and high precision [5]. USG-based normative data for laryngeal structures need to be established, as they can be valuable for both diagnostic and therapeutic purposes [2]. The present study aimed to examine a USG-assisted critical vocal fold biomarker, the glottal area. It also investigated the association between the glottal area and acoustic parameters, including *f*_0_, jitter, and CPP. Additionally, the study explored the correlation of the glottal area with USG-derived measures of VFL and VFDV, as well as its relationship with age and gender. In healthy participants, vocal fold morphology was assessed using anatomical landmarks, including the thyroid cartilage, arytenoid cartilage, false vocal folds, true vocal folds, cricoid cartilage, and the anterior and posterior commissures. These landmarks were well visualized across all healthy individuals, aligning with earlier findings on the evaluation of vocal structures in normophonic adults [31]. Vocal fold symmetry was evaluated based on vocal fold mobility, with all participants exhibiting perfect symmetry (Grade I). Similar findings have been reported in earlier research, where symmetrical vocal fold movements were observed both before and after thyroid surgery. In that study, patients were classified as ‘Grade I’ preoperatively, reflecting normal symmetry, while postoperative assessments showed classifications of Grade II and III, indicating reduced or absent vocal fold movement [32].

The present study aimed to establish norms for glottal area measured using USG during the inspiratory phase of QB. Glottal area analysis has proven effective in detecting certain laryngeal diseases and evaluating therapy progress [20]. In the present study, the mean glottal area during the inspiratory phase of QB was measured using USG, yielding values of M = 1.407 cm² (SD = 0.086 cm²) for males and M = 1.106 cm² (SD = 0.080 cm²) for females. These findings are consistent with previous studies that have utilized various imaging techniques to assess glottal dimensions during QB. For instance, a study [5] investigated glottal area in healthy adults using USG where authors examined the vocal folds using real-time video clips, from which snapshots were extracted for analysis. Findings reported a mean (M) area minimum of 0.37 cm² (SD = 0.18 cm²) and a mean (M) area maximum of 1.01 cm² (SD = 0.26 cm²) in their control group which consisted of healthy volunteers [5]. Although the values obtained in our study are slightly higher, especially for males, they remain within a comparable range, suggesting that our glottal area values are consistent with previous research. The minor differences could be attributed to demographic variations or methodological factors, such as the specific imaging plane and frame selection criteria. In this study the glottal area was analysed using still images selected at the point of full vocal fold abduction during inhalation.

Another finding in the present study was that males demonstrated a significantly larger mean glottal area, during the inhalation phase of QB, compared to females. This aligns with previous research reporting greater glottal area measurements in males [18]. The glottal area in the current study was calculated using the formula (½ × base × height), based on prior methodologies [29]. Males generally have longer vocal folds than females, a difference supported by previous research [1, 18] and reaffirmed by the current study. This longer vocal fold length among males is generally attributed to anatomical differences and the influx of testosterone during puberty, resulting in larger vocal fold muscles, increased mass, and prominent thyroid structures in males [26, 33]. Given the method used to estimate glottal area (½ × base × height) [29], the longer, thicker vocal folds in males could be producing greater vocal fold excursions and larger amplitudes, resulting in greater glottal area [26]. This relationship gets further reinforced evident in the present study, where a significant strong positive correlation between VFL and glottal area was observed in males, consistent with previous findings [18].

Furthermore, the study revealed significantly higher VFDV values in females than in males across all conditions and sides, a finding consistent with [1], who also reported greater VFDV in females using USG. These differences are likely attributed to anatomical and physiological variations such as shorter and lighter vocal folds in females, leading to quicker displacement during oscillation [1, 19]. Additionally, a strong, statistically significant negative correlation was observed between glottal area and VFDV on both the right and left sides suggesting that larger glottal areas and longer VFL’s, commonly seen in males, may be associated with reduced displacement velocity. Increased tissue mass resulting from longer vocal folds in males has been associated with a reduction in the number of vocal fold oscillations, as evidenced by decreased frequency [34, 35]. With the negative correlation observed between the glottal area and the VFDV, it would be therefore reasonable to assume that greater tissue mass and inertia not only lower frequency but may also contribute to a reduction in VFDV [35, 36]. While this relationship has not been extensively reported in the literature, it aligns with biomechanical principles governing vocal fold oscillation [35, 36] and invites further investigation.

The study focused on young normophonic adults (aged 20–35 years) to examine whether age-related changes in glottal area could be detected within this group. No significant relationship between USG-aided glottal area measurements and age was found in both males and females. These findings are representative of this specific age group and align with [18], who also reported no significant glottal configuration differences within this range, despite age-related changes observed in older adults. This suggests that individuals in this age group exhibit relatively homogeneous glottal characteristics, and the data obtained can be considered applicable to the entire age range of young normophonic adults.

A significant positive correlation (*p* = 0.025) between glottal area and CPP with voice detection observed exclusively during/i/phonation suggests that as glottal area increases there is a better harmonic-noise characteristic as measured by CPP. This finding may be attributed to the distinct vocal tract configuration associated with this vowel. The high front tongue position and increased supraglottic impedance during/i/are known to enhance glottal excitation and acoustic feedback, promoting more periodic vocal fold vibration [37]. Consequently, increases in glottal area may still correspond to a highly periodic voice signal, yielding higher CPP values. In contrast, the more open configuration of/a/reduces vocal tract impedance, potentially increasing acoustic noise and signal aperiodicity, which may obscure any relationship between CPP and glottal area [37]. Among the acoustic parameters, *f*_0_, jitter, and CPP (for both/a/and/i/phonation), as well as CPP with voice detection during/a/phonation, showed no significant correlation with glottal area. This may be attributed to the differing contexts in which the measurements were taken, acoustic parameters were extracted during phonation, while glottal area estimation in the present study was conducted during QB. These contextual differences could have influenced the lack of correlation. Therefore, future research should explore whether glottal area estimation during phonation shows a stronger relationship with acoustics measured in the same context.

This study establishes normative data for glottal area estimates in young normophonic individuals. Since glottal area tends to vary in cases of mass occupying lesions or physiological impairments such as vocal fold paralysis, where a reduction in glottal area is often noted, these values may serve as useful clinical reference points when assessed through USG. Building on prior research that identified VFL and VFDV as promising USG-based biomarkers, this study adds glottal area as another significant parameter, enhancing the utility of USG in vocal fold assessment.

## Conclusion

USG emerges as a promising, non-invasive, and cost-effective method for assessing laryngeal function. While previous studies have explored parameters such as VFL and VFDV, the present study adds to this body of knowledge by incorporating glottal area measurements. Glottal area measurement, when evaluated using USG, offers a valuable and objective means of assessing laryngeal function. Although the lack of established normative data has limited USG’s widespread clinical application, this study provides baseline USG-aided glottal area values for young adults, offering a valuable reference point for clinical evaluations. These findings support the use of USG as an effective tool for SLPs in diagnosing and managing voice disorders.

## Clinical implications

The normative glottal area values obtained through USG in this study can serve as a reference for identifying normal vocal fold function in young normophonic adults, aiding in the diagnosis of voice disorders. This data also holds clinical relevance for monitoring therapeutic outcomes, for instance, assessing whether decrease in mass lesions or improvements in vocal fold mobility (e.g., in paralysis) correspond to proportional changes in glottal area. USG-based glottal area estimation thus offers a valuable, objective tool for tracking vocal fold status during intervention, supporting more tailored and effective treatment plans. These findings represent an important step toward broader clinical usage of USG, previously limited by the absence of normative data.

## Limitations

The present study assessed glottal area only during QB, specifically during the inhalation phase; measurements during phonation and the exhalation phase were not conducted. Additionally, the sample had an unequal distribution of male and female participants, which should be addressed in future research. Acoustic analysis was performed on only a small subset of participants due to logistical constraints.

While the study reports consistent visualization of anatomical landmarks across participants using USG, cervical ultrasound imaging has known limitations, most notably, interference caused by laryngeal cartilage calcification [38], a phenomenon particularly common in older populations [39]. Although this was not a concern in the present study involving young normophonic adults, it may pose challenges when applying this method to older populations. Such calcification could degrade image quality, hinder interpretation, and ultimately limit clinical utility [38]. In such cases, future studies could explore contingency measures such as switching to laryngoscopy in cases of poor visualization.

Lastly, intra and inter-observer reliability assessments for USG-derived measurements such as VFL, VFDV and glottal area were not conducted in this study, which limits the ability to evaluate the reproducibility of the findings. A logical next step would be to compare USG-derived data with simultaneously obtained laryngoscopic frames to validate consistency and guide standardization. Additionally, comparing readings from different examiners could help establish reliability.

## Future directions

Future research could extend USG-based assessments of glottal area, VFL and VFDV to broader age groups, including middle-aged and older adults, as well as individuals with various vocal pathologies. To ensure better population representation, larger sample sizes will be essential. Incorporating vocal loudness monitoring during USG evaluations may offer additional clinical insights. Moreover, establishing normative data across age groups will help clarify age-related vocal trends. Finally, the development of a standardized USG training protocol is crucial to minimize operator variability and enhance consistency in interpretation.
